# Epichromatin and chromomeres: a ‘fuzzy’ perspective

**DOI:** 10.1098/rsob.180058

**Published:** 2018-06-06

**Authors:** Donald E. Olins, Ada L. Olins

**Affiliations:** Department of Pharmaceutical Sciences, College of Pharmacy, University of New England, 716 Stevens Avenue, Portland, ME 04103, USA

**Keywords:** histones, nucleosomes, intrinsically disordered regions, avidity

## Abstract

‘Epichromatin’, the surface of chromatin beneath the interphase nuclear envelope (NE) or at the surface of mitotic chromosomes, was discovered by immunostaining with a specific *bivalent* mouse monoclonal anti-nucleosome antibody (mAb PL2-6). ‘Chromomeres’, punctate chromatin particles approximately 200–300 nm in diameter, identified throughout the interphase chromatin and along mitotic chromosomes, were observed by immunostaining with the *monovalent* papain-derived Fab fragments of bivalent PL2-6. The specific target for PL2-6 appears to include the nucleosome acidic patch. Thus, within the epichromatin and chromomeric regions, this epitope is ‘exposed’. Considering that histones possess unstructured ‘tails’ (i.e. intrinsically disordered peptide regions, IDPR), our perception of these chromatin regions becomes more ‘fuzzy’ (less defined). We suggest that epichromatin cationic tails facilitate interactions with anionic components of NE membranes. We also suggest that the unstructured histone tails (especially, histone H1 tails), with their presumed promiscuous binding, establish multivalent binding that stabilizes each chromomere as a unit of chromatin higher order structure. We propose an ‘unstructured stability’ hypothesis, which postulates that the stability of epichromatin and chromomeres (as well as other nuclear chromatin structures) is a consequence of the *collective* contributions of numerous weak histone IDPR binding interactions arising from the multivalent nucleosome, analogous to antibody avidity.

## 1. Background and recent data

Although the nucleosome was discovered more than 40 years ago [1], the next level of organization, the higher order chromatin structure, remains controversial. We believe this is due to the search for a rigid organization in a highly dynamic and fluid nuclear architecture. This review will focus upon chromatin architecture at the nuclear envelope (NE), at the surface of mitotic chromosomes and, to some extent, chromatin within nuclei and mitotic chromosomes. Interphase NE chromatin, its association with inner nuclear membrane proteins, mitotic chromosome structure and post-mitotic NE reformation are discussed within numerous excellent recent articles [2–11]. This personalized brief review will primarily discuss recent discoveries from our laboratory and their possible implications to the larger issue of chromatin higher order structure.

**Figure 1.**
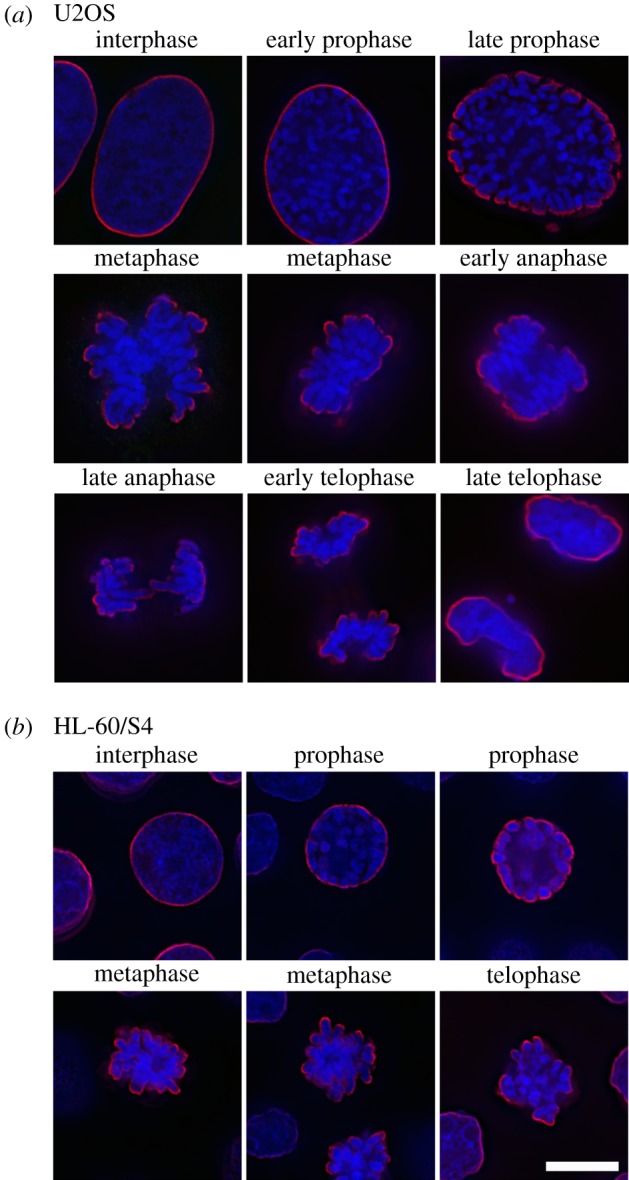
Immunostaining of the epichromatin epitope throughout the cell cycle in U2OS (*a*) and HL-60/S4 (*b*) cells using mAb PL2-6 (red) and a DNA stain (DAPI, blue). Note that epichromatin staining persists on the outer edges of the mitotic chromosomes, even following NE breakdown. The magnification bar for (*a*) and (*b*) equals 10 µm. This image has been previously published [15].

**Figure 2.**
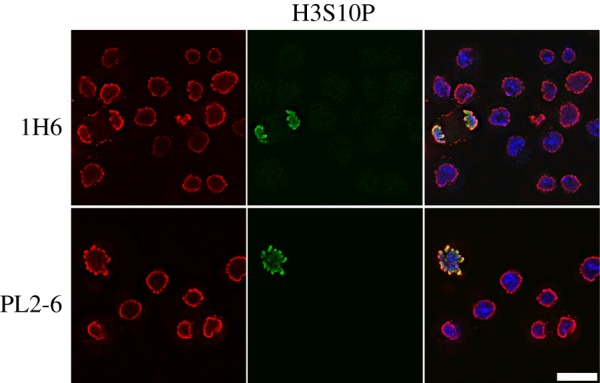
Immunostaining of the epichromatin epitope in interphase and mitotic *Drosophila* Kc cells using two different mAbs that stain epichromatin (PL2-6 and 1H6, red), rabbit polyclonal anti-H3S10p (green) and DNA (DAPI, blue). Note the similar staining of epichromatin as shown for human U2OS and HL-60/S4 cells (figure 1). The magnification bar equals 10 µm. This image has been previously published [16].

**Figure 3.**
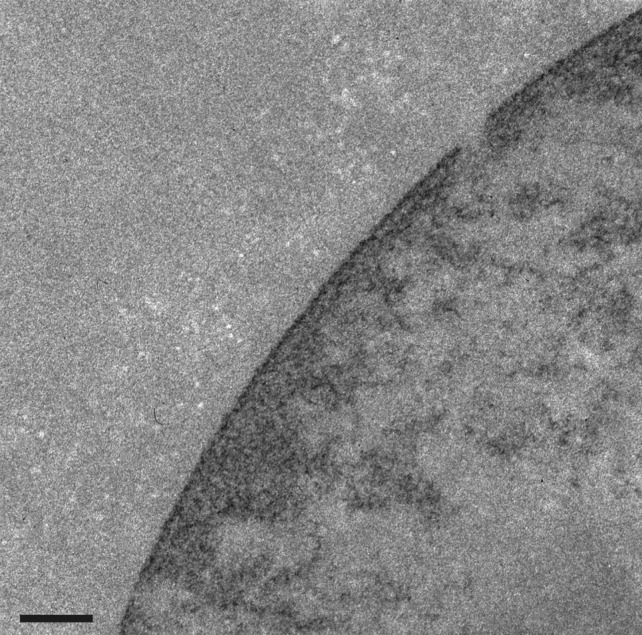
DNA-specific staining (OAB) of an interphase HL-60/S4 cell imaged by thin section transmission electron microscopy. OAB shows a region of intense staining at the periphery of the interphase nucleus, suggesting a higher concentration of ordered DNA in the epichromatin region. The magnification bar equals 0.2 µm.

**Figure 4.**
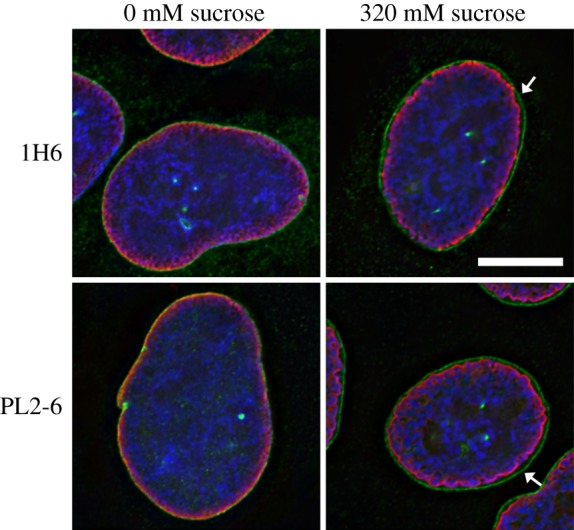
Immunostaining of the epichromatin epitope in interphase U2OS cells exposed to hyper-osmotic conditions (320 mM sucrose), compared to iso-osmotic conditions (0 mM sucrose), employing two different mAbs that stain epichromatin (PL2-6 and 1H6, red), two rabbit antibodies that stain NE proteins (emerin and lamin A, green) and a DNA stain (DAPI, blue). The arrows point to a gap between the NE (lamina) and epichromatin, observed in 320 mM sucrose. The magnification bar equals 10 µm. This image has been previously published [16].

Antibodies against nuclear components and structures are a common feature of many autoimmune diseases, yielding important reagents in the study of interphase nuclei and mitotic chromosomes [12,13]. Indeed, the principal antibody tool (mAb PL2-6) of our laboratory for the past few years was discovered in the laboratory of Marc Monestier, during an analysis of mouse autoimmunity [14]. PL2-6 was developed into a hybridoma along with other anti-histone antibodies. When several of these antibodies were examined in our laboratory, we observed that PL2-6 produced a remarkably similar immunostaining pattern on cells from a variety of diverse species (human, mouse, *Drosophila*, *Caenorhabditis elegans* and tobacco) [15,16]: interphase nuclei exhibited strong staining beneath the NE, denoted ‘epichromatin’; mitotic chromosomes frequently exhibited even stronger staining at the outer edges of the chromosome surfaces (figures 1 and 2). The intense peripheral staining of the interphase nuclei was observed using thin section electron microscopy of interphase HL-60/S4 cells treated with the DNA-specific osmium ammine B (OAB) stain [17–19]; an example is shown in figure 3. This OAB stained image suggests that the DNA may be more densely packed just beneath the NE. More recently, we demonstrated that exposing interphase mammalian tissue culture cells to hyper-osmotic conditions (i.e. 320 mM sucrose in culture medium) resulted in contraction of interphase chromatin away from the NE, clearly revealing that the epichromatin epitope is separate from the NE lamina (figure 4).

**Figure 5.**
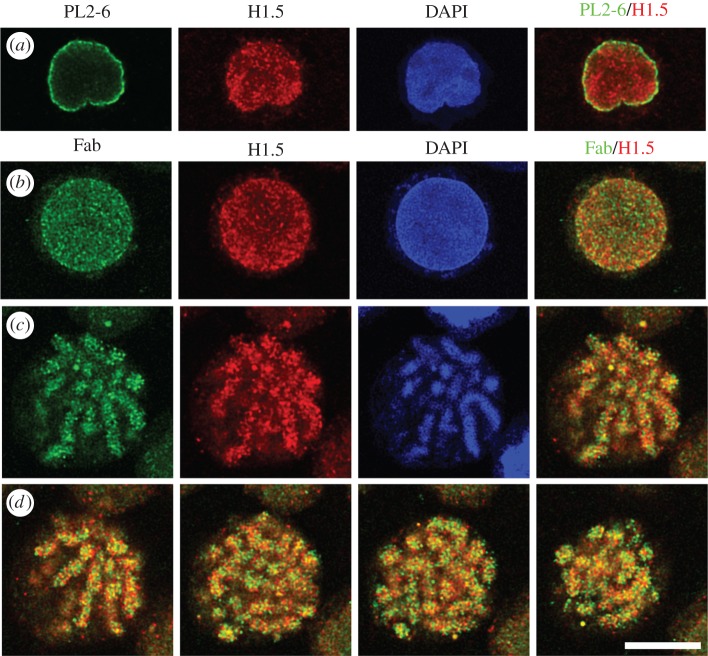
Immunostaining patterns of bivalent PL2-6 and monovalent Fab fragments, derived from PL2-6. (*a*) An undifferentiated HL-60/S4 interphase nucleus stained with PL2-6 (green), anti-histone H1.5 (red), DAPI (blue) and merged red and green (R + G). (*b*) An undifferentiated HL-60/S4 interphase nucleus stained with Fab (green), anti-histone H1.5 (red), DAPI (blue) and merged (R + G). (*c*) A single confocal Z-slice from a mitotic HL-60/S4 cell stained with Fab, anti-histone H1.5, DAPI and the merged (R + G). (*d*) Various Z-slices from the merged mitotic R + G stack are presented. For all images, the magnification bar equals 10 µm. This image has been previously published in part [20].

**Figure 6.**
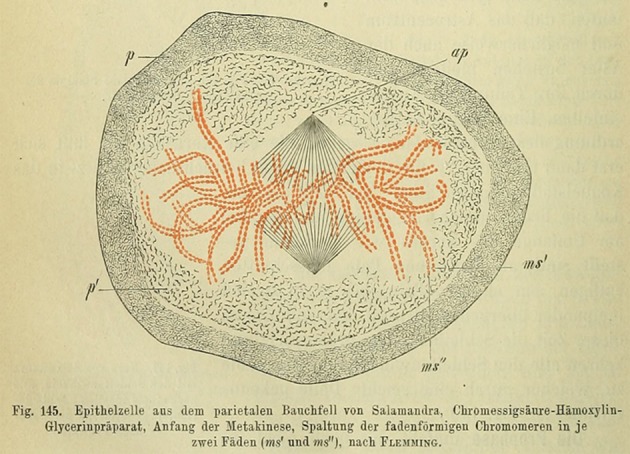
Mitotic figure from a stained salamander epithelial cell, drawn by Walther Flemming, originally published in ‘Zellsubstanz, Kern und Zelltheilung’ (1882, Tafel IIIb, Fig. 41). In the figure caption, Flemming specifically noted the chromosome substructure ‘Körnelung sehr deutlich’ (granulation very distinct). In 1896, this drawing was reprinted by H. Fol [22], who is generally credited with introducing the term ‘chromomere’. However, in his own caption to the reprinted figure, he appears to be describing the chromatid fibres not the chromatin granules. Simultaneously [23], in his classic book ‘The Cell in Development and Inheritance’ (1896), E. B. Wilson wrote in Chapter VI, p.221 the following: ‘The facts are now well established (1) that in a large number of cases the chromatin-thread consists of a series of granules (chromomeres) embedded in and held together by the linin-substance, (2) that the splitting of the chromosomes is caused by the division of these more elementary bodies … . These facts point unmistakably to the conclusion that these granules are perhaps to be regarded as independent morphological elements of a lower grade than the chromosomes.’ It is in that spirit that we have denoted our immunostained chromatin granules as ‘chromomeres’.

**Figure 7.**
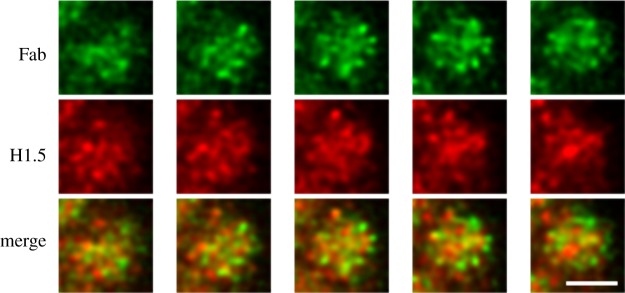
Immunostaining patterns of monovalent Fab fragments on alternate sequential Z-slices along a mitotic chromosome arm, revealing a radial ‘chromomeric’ pattern. The top row is Fab (green); middle row, anti-histone H1.5 (red). The bottom row contains the merged ‘red + green (R + G)’ slices. Chromomeres are approximately 300 nm in diameter. The magnification bar equals 2 µm. This image has been previously published [20].

**Figure 8.**
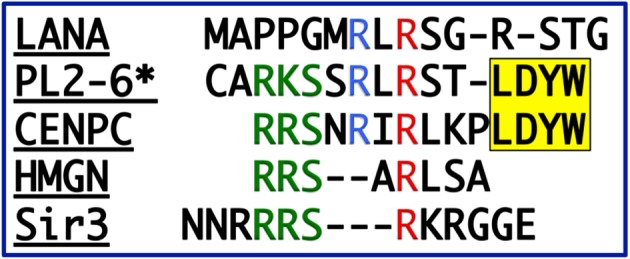
Peptide sequence comparisons. The PL2-6 heavy chain variable region 3 (‘*’, Hv3) compared to various acidic patch binding proteins. The second arginine (red R) in the LANA sequence (… RLRS …) forms salt bridges with H2A E61, D90 and E92 [24]. The yellow (… LDYW …) motif is a common hydrophobic structural feature of many Hv3 regions. This image has been previously published [20].

PL2-6 is a bivalent mouse IgG2b antibody. In order to examine the significance of antibody bivalency to the remarkable peripheral chromatin nuclear staining, we generated a monovalent form of PL2-6, employing papain digestion [20]. To our surprise, the monovalent Fab fragments yielded a punctate immunostaining pattern throughout interphase nuclei and along mitotic chromosome arms (figure 5). These (formaldehyde-fixed) punctate structures are approximately 200–300 nm in diameter and have been named ‘chromomeres’ [20], in deference to the pioneering observations of mitotic chromosome granules observed at the end of the nineteenth century [21–23] (figure 6). In favourable microscopic views, chromomeres appear to radiate out of a central region in the chromosome arms (figure 7). During the initial discovery of mAb PL2-6 [14], the authors demonstrated that histones H2A and H2B include the epitope site. By comparison of the peptide sequence of the PL2-6 (heavy chain variable region 3, hv3) to those of known nucleosome ‘acidic patch’-binding proteins (LANA and CENP-C), we predicted that the acidic patch (an ‘exposed’ juxtaposition of acidic amino acids in histones H2A and H2B) would include the epitope (figure 8) [20].

Several major questions arise from the unusual chromatin surface binding of interphase epichromatin by bivalent PL2-6: What are the properties of this chromatin? Do these properties change during cell differentiation? What type of DNA is present in interphase epichromatin? These questions were explored employing ChIP-Seq on the human myeloid leukaemia HL-60/S4 cell line, which can be differentiated *in vitro* into granulocytes and macrophage forms [25]. In summary, ChIP-Seq revealed considerable similarity in the DNA composition comparing interphase epichromatin from undifferentiated and differentiated cells. Epichromatin contains only approximately 4–5% of the total DNA sequences. In the HL-60/S4 cells, it is GC-enriched, highly methylated and exhibits a significant enrichment in retrotransposon Alu. Mapping epichromatin regions along the human chromosomes demonstrated considerable similarity in its discrete and discontinuous distribution, comparing the three cell states. Furthermore, epichromatin exhibits a high nucleosome density and a *paucity* of various histone post-translational modifications associated with transcription or repression of transcription [26]. Indeed, it appears that interphase epichromatin represents a unique unmodified (except for DNA methylation) chromatin ‘surface’ facing the inner membrane of the existing (or reforming) NE. It is important to stress that PL2-6 is *not* an anti-DNA antibody and that the DNA sequences enriched from HL-60/S4 cells are possibly unique to these cells. Certainly, *Drosophila*, *C. elegans* and tobacco cells do not have retrotransposon Alu, among other differences. A recent study [27], employing acridine orange staining, argues that epichromatin DNA possesses an A-form conformation more readily than internal chromatin.

Much less can be said about chromomeres, visualized with the monovalent Fab fragments of PL2-6. Chromomeres appear to have ‘exposed’ nucleosome acidic patches which are not highlighted by bivalent PL2-6. It is possible that some chromomeres present transcriptional ‘target regions’ on their surface, maintaining a more ‘open’ display of surface nucleosomes [28], but we have no information on the relationship of chromomeres to transcription. Chromomeres can also be visualized using bivalent anti-H1 antibodies [20], which implies that chromomere surfaces exhibit ‘exposed’ H1 epitopes. There appears to be some level of chromomere heterogeneity, with some chromomeres exhibiting particular H1 isotypes and others showing absence of particular H1 isotypes. Present immunostaining data are not sufficient to say whether chromomeres contain more than one H1 isotype. HL-60/S4 cells (undifferentiated and differentiated) have been demonstrated to contain primarily three H1 isotypes, H1.2, H1.4 and H1.5 [29]. Depending upon image ‘thresholding’ constraints, the number of HL-60/S4 Fab-stained undifferentiated interphase chromomeres (per diploid nucleus) is approximately 2791 (at 20% threshold) and less (approx. 1150) in mitotic chromosomes [20]. It is not clear whether this apparent reduction in chromomeres during metaphase is due to chromatin conformational changes or to the epichromatin epitope becoming ‘hidden’, or both. It is clear that loops in mitotic chromosomes, whether nested or tightly packaged, are highly condensed [11] and could appear as chromomeres in formaldehyde-fixed chromosomes.

**Figure 9.**
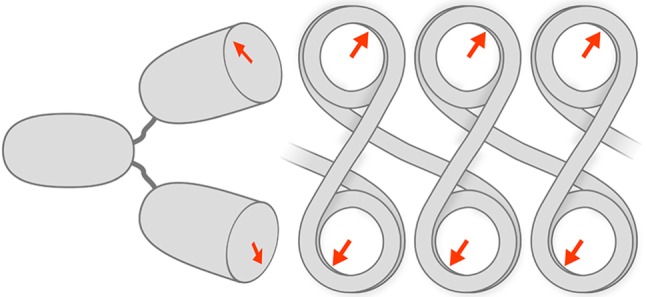
Cartoon sketch depicting a bivalent PL2-6 molecule binding to a zig-zag arrangement of nucleosomes. This sketch does not display accurate molecular dimensions. Rather, it is exaggerated to show the potential sites of interaction. A more accurate model can be visualized in the electronic supplementary material, Movie. The cartoon arrows on the nucleosome ‘faces’ represent the nucleosome acidic patches (AP). Notice that in this zig-zag arrangement, for sequential nucleosomes, the AP orientations are related by a dyad axis (perpendicular to the linker DNA and to the plane of the cartoon). Adjacent to the zig-zag chromatin is a representation of the bivalent PL2-6, also displaying the complementary binding regions as arrows. A bivalent IgG has a dyad axis running between the two Fab regions and along the midline of the Fc region. The junction between the Fab and Fc regions is very flexible, tolerating a wide range of angles between the Fab ‘arms’ and allowing the arms to rotate with considerable freedom. The geometry of the nucleosomes presents two binding sites to the bivalent IgG, exploiting the avidity principle.

How do we explain the different immunostaining patterns of bivalent PL2-6 and of monovalent Fab fragments? Conceivably, at least four factors might affect the bivalent versus the monovalent antibody staining pattern: (i) epitope local concentration; (ii) geometric arrangements of the multivalent epitopes; (iii) blocking by competing binders; and (iv) unique properties of epichromatin as a ‘surface’ (e.g. chromatin on one side; no chromatin on the other side). It is interesting that a zig–zag arrangement of nucleosomes along a single chromatin fibre (a view quite common in surface spread chromatin fibres; see [1]) yields adjacent nucleosomes exhibiting acidic patches related by an external dyad axis, which could match complementary bivalent PL2-6 binding sites (also related by an internal dyad axis; figure 9 and the electronic supplementary material, Movie). We suggest that the intense localized immunostaining pattern of PL2-6 is an example of high antibody ‘avidity’ (i.e. the binding of a bivalent antibody to a multivalent antigen, resulting in an increased ‘association constant’) binding to ‘exposed’ epitopes present at a high local concentration [30]. The monovalent Fab fragment does not have the property of avidity (with its high association constant) and is less dependent upon local geometry. But it is still influenced by epitope concentration and blocking by competitors. In sum, we suggest that bivalent PL2-6 is greatly responsive to the nucleosome–nucleosome geometry at the epichromatin surface, benefiting by its capability for avidity. The monovalent Fab can detect ‘exposed’ epitopes scattered throughout the nuclear and chromosome environment, revealing clustered epitopes defining the punctate chromomeres.

## 2. Discussion

The 1950s and 1960s were the beginning of exciting times for structural biology: deduction of the protein α-helix and β-sheet from X-ray diffraction data of polypeptides, determination of the collagen triple helix, the DNA double helix, haemoglobin subunit allosteric interactions, predictability of RNAse peptide folding, tRNA structure, etc. In many respects, the cell became regarded as a ‘bag’ of highly regular macromolecular structures, functioning by ‘lock-and-key’ mechanisms with predictable secondary, tertiary and quaternary higher order folding, while being immersed in defined intracellular solutions. An amazing documentation of the successes with structure determination can be seen in the beautiful poster available online from the PDB (Molecular Machinery: A Tour of the Protein Data Base). This confidence in structural predictability, combined with fibre X-ray diffraction data (i.e. the method employed to decipher the DNA double helix) employed on isolated chromatin fibres led to two proposals for helical models of chromatin, both of which were wrong (aspects of this part of chromatin history, viewed through the eyes of the present authors, have been previously published) [1]. It is now clear that much of cellular structure, including chromatin, is more complex and disorganized than we had hoped. Soon after the discovery of the nucleosome in 1973/1974, helical arrangements of nucleosomes into approximately 30 nm fibres became the favoured next level of chromatin organization. Numerous studies, including electron microscopy and X-ray scattering, have eroded our confidence in the general existence of such structures [31–36]. Instead, current views emphasize less well-defined punctate super-nucleosomal clusters (with various names; e.g. fractal globules, topologically associating domains, contact domains, compact domains and 1 Mbp chromatin domains) [37–44], structures that we suggest are correlates with our fixed ‘chromomeres’ [20]. These structures undoubtedly reflect an underlying genetic organization and are reminiscent of the earlier studies of lampbrush and polytene chromosomes [45–49].

Describing the internal nucleosomal organization of chromomeres presents a distinct challenge. Histone H1 is a particularly attractive candidate for stabilizing super-nucleosomal clusters (chromomeres) within live cells. A considerable amount of research has been performed on the different H1 isotypes (generally, six isotypes in somatic human cells: H1.1, H1.2, H1.3, H1.4, H1.5 and H1.0 in terminally differentiated cells) [50–62]. A few of the conclusions presented in these many research and review articles are as follows. (i) A central highly conserved globular domain with a ‘winged helix fold’ [63], which binds to the nucleosome, is flanked by two unstructured peptide tails. (ii) *In vitro*, the presence of histone H1 is required to condense polynucleosomal chains at physiological ionic strength. (iii) The H1 C-terminal peptide is more important in the formation of chromatin higher order structure than is the N-terminal peptide (which still plays a role). (iv) There appears to be an insignificant amount of ‘free’ (unbound) H1 in the nucleus. (v) The residence time for H1 on a nucleosome is shorter than the residence time for the inner histones, but longer than for transcription factors or HMG proteins. (vi) H1 exhibits numerous types of post-translational modifications (especially phosphorylation) that are dynamic during the cell cycle and during cell differentiation. (vii) Genetic loss of certain histone isotypes can apparently be compensated by H1 redundancy, until the stoichiometry of H1/nucleosome becomes too low. It is clear that H1 proteins are dynamic within the nucleus, with complex and varying roles in influencing chromatin higher order structure and transcription regulation.

**Figure 10.**
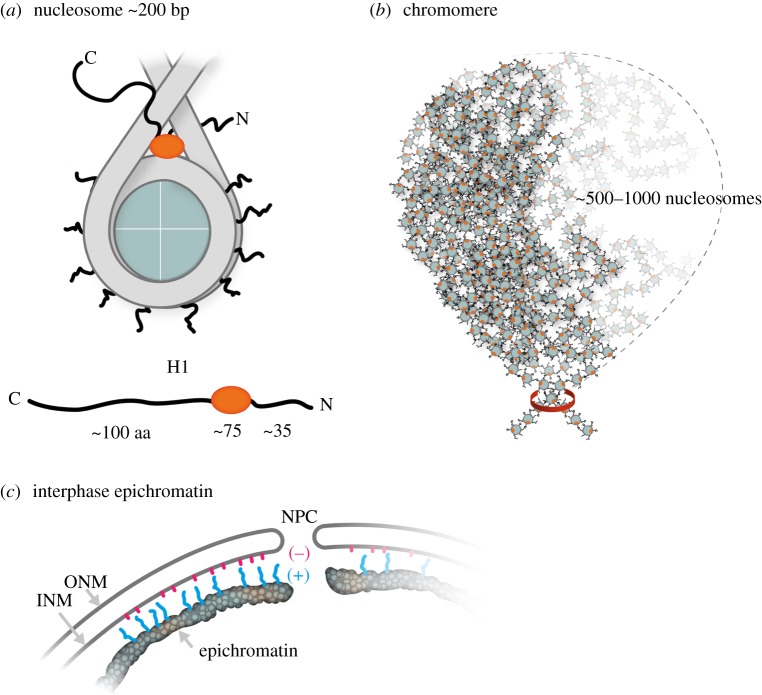
Cartoons depicting locations of intrinsically disordered peptide regions (IDPR) at three levels of chromatin structure, the nucleosome, the chromomere and epichromatin. (*a*) Cartoon of a mononucleosome with 10 basic inner histone tails and two basic histone H1 tails forming a multivalent macromolecule with possible analogy to the multivalent IgM antibody molecule. Beneath the nucleosome is a drawing of an H1 molecule, showing the central globular region (red) flanked by N- and C-terminal tails. An untethered 100 aa peptide can extend up to approximately 30–35 nm from the globular domain. (*b*) Cartoon of a chromomere, contained within a membrane-less domain (approx. 200–300 nm diameter), consisting of a highly convoluted polynucleosome chain (possibly, approximately 500–1000 nucleosomes [42,71]) clamped by condensin or cohesin (red ring) [72,73] into a genetic ‘bouquet’, stabilized by the promiscuous binding of histone tails to adjacent nucleosomes, resulting in a spatially coordinated higher order structure. (*c*) Cartoon of interphase epichromatin adjacent to the NE, containing a nuclear pore complex (NPC), an outer nuclear membrane (ONM) and an inner nuclear membrane (INM). The outermost layer of nucleosomes in epichromatin exhibits ‘waving’ (+) charged histone tails (blue) interacting with the anionic (−) phospholipids (red) emanating from within the INM. This cartoon does not show the lamins nor transmembrane proteins (e.g. LBR, emerin, etc.), but focuses on the epichromatin regions in contact with the INM.

An additional challenge to the concept of highly defined chromatin structures comes from the emerging realization that the constituent histones of the nucleosome are rich in ‘unstructured’ intrinsically disordered peptide region (IDPR) [64–66]. IDPRs are frequently enriched with Lys, Arg, Pro and Ser residues, a clear characteristic of histone tails. IDPRs, by definition, do not exhibit stable peptide conformations in physiological buffers and are generally promiscuous in their interactions with binding partners. IDPR conformations are greatly affected by post-translational modifications [67] and usually acquire more defined peptide conformations as a consequence of binding [68]. Because of the frequently chaotic nature of the IDPR conformations in solution, they have been aptly described as ‘fuzzy’ or forming a peptide ‘cloud’ [69]. Nucleosomes possess 10 inner histone IDPR tails [65] and two IDPR histone H1 tails, becoming multivalent structures with numerous types of binding interactions [43,70] (figure 10*a*). As an analogue to the multivalent mononucleosome, we suggest considering the multivalent IgM (macro-immunoglobulin) molecule. The IgM molecule is composed of five IgG-like subunits, connected by their Fc regions, generating 10 antibody binding sites on a single macromolecule. The individual binding constants are usually relatively weak; but collectively, based upon the avidity principle, an IgM molecule has among the strongest association constant of any immunoglobulin. As a prototype for inter-nucleosome binding, we also cite the histone H4 tail, which can interact with the acidic patch of an adjacent nucleosome [74]. We suggest that ‘fuzzy’ nucleosomes form the basis for ‘fuzzy’ epichromatin and ‘fuzzy’ chromomeres. The unstructured basic tails extending outward from epichromatin could form electrostatic interactions with nuclear membrane anionic phospholipids (e.g. phosphatidylserine, PS) (figure 10*c*), analogous to the interaction between PS and the protein MARCKS at the plasma membrane [75,76]. The inner histone and H1 tails could be binding to neighbouring nucleosomes helping to stabilize the chromomeric nucleosome complex, acting as promiscuous ‘sticky tape’ (figure 10*b*). In this manner, nuclear architecture and chromatin higher order structure can be regarded as being the product of the multivalent mononucleosome, with its binding force derived from the avidity principle.

## 3. Speculations

(1) We suggest that the conformational plasticity of nucleosomal higher order structure is in large measure due to the multiplicity of IDPR histone tail interactions.
(2) Our conception of interphase and mitotic epichromatin is of a chromatin surface covered with unaffiliated histone tails, forming a cloud of (+) amino acid residues. Interphase epichromatin might be electrostatically attracted to inner nuclear membrane anionic phospholipids. During mitotic chromosome condensation, a rise in cellular Mg^2+^ [77] might weaken the electrostatic interaction of the histone tails to the membrane anionic phospholipids. During telophase, the positively charged chromatin surface may facilitate post-mitotic NE reformation.
(3) We speculate that inner histone and H1 tails act like ‘sticky tape’ holding multivalent nucleosomes in a coordinated bundle (chromomeres). The effectiveness of this ‘sticky tape’ to bind nucleosomes together can be influenced by post-translational modifications. In vitro experimental and computational evidence supports that the histone tails can bind to DNA within chromatin [78–80]. Thus, it is likely that *in vivo*, at least some of the histone tails are associated with ‘distant’ nucleosomal DNA.
(4) Chromomeres likely represent only one level in the super-nucleosome organization. If chromatin possesses ‘liquid droplet’-like properties [41], phase separation may fuse together these structures into larger structures or cleave them into smaller units.

## 4. The ‘unstructured stability’ hypothesis

We propose that various chromatin structures (e.g. epichromatin and chromomeres) are stably maintained by the interactions of the unstructured intrinsically disordered peptide regions (IDPRs) of the inner histones and of histone H1 on multivalent mononucleosomes. This hypothesis stresses the *collective* contributions of the numerous IDPR–binding partner interactions in establishing these chromatin structures. Furthermore, we propose that the promiscuity of IDPRs will generate many functionally equivalent binding partners (e.g. consisting of DNA, protein or phospholipids), resulting in considerable redundancy, such that mutations in the binding sites can be readily compensated by alternative binding interactions. However, this hypothesis must be consistent with the conception that histone post-translational modifications of IDPRs can modulate (i.e. destabilize or hyper-stabilize) these binding interactions. This hypothesis also does not exclude the importance of specific ‘lock-and-key’ interactions by other chromatin-associated proteins or specific ‘induced fit’ mechanisms by modifying enzymes [81], which may be essential for the specific genetic functions. Rather, this hypothesis focuses attention upon the considerable number of weak and relatively nonspecific interactions, in combination with the avidity principle, that permit chromosomes to fulfil their numerous functions. Testing the hypothesis will require cataloguing and mutating the multitude of IDPR binding interactions, a considerable endeavour. From the point-of-view of Darwinian evolution, if the Unstructured Stability Hypothesis is correct, one could argue that a preservation of some level of IDPRs is advantageous for maximizing evolutionary adaptability.

## Supplementary Material

Possible binding of Antibody PL2-6 to a Dinucleosome

## Data accessibility

This article has no additional data.
