# Supplementary material for: Epichromatin and chromomeres: a ‘fuzzy’ perspective
Source: Open Biol. 2018 Jun 6;8(6):180058. doi: 10.1098/rsob.180058 (PMC6030114; doi:10.1098/rsob.180058)

## Slide 1
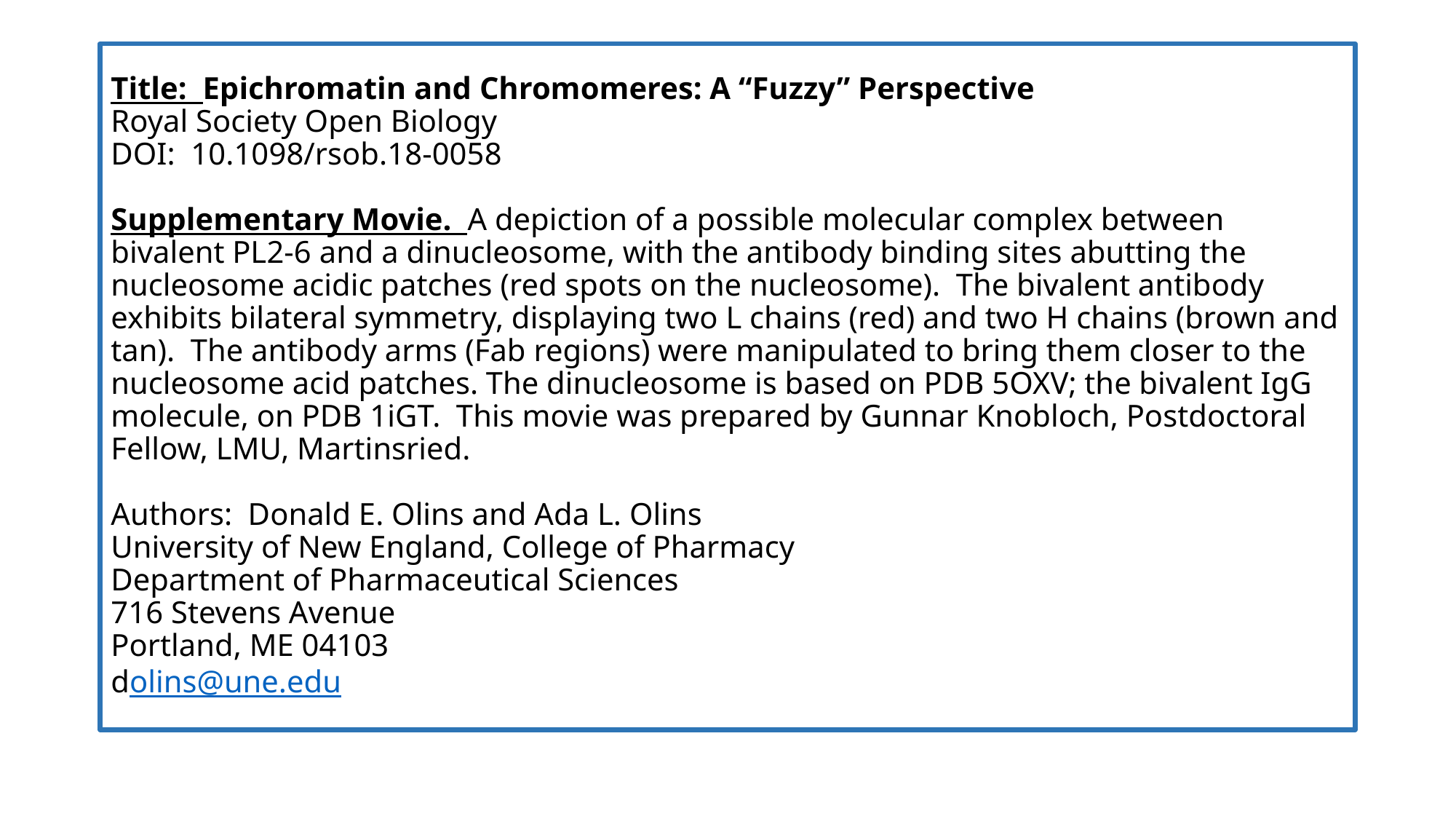

# Title: Epichromatin and Chromomeres: A “Fuzzy” Perspective Royal Society Open Biology DOI: 10.1098/rsob.18-0058 Supplementary Movie. A depiction of a possible molecular complex between bivalent PL2-6 and a dinucleosome, with the antibody binding sites abutting the nucleosome acidic patches (red spots on the nucleosome). The bivalent antibody exhibits bilateral symmetry, displaying two L chains (red) and two H chains (brown and tan). The antibody arms (Fab regions) were manipulated to bring them closer to the nucleosome acid patches. The dinucleosome is based on PDB 5OXV; the bivalent IgG molecule, on PDB 1iGT. This movie was prepared by Gunnar Knobloch, Postdoctoral Fellow, LMU, Martinsried. Authors: Donald E. Olins and Ada L. Olins University of New England, College of Pharmacy Department of Pharmaceutical Sciences 716 Stevens Avenue Portland, ME 04103 dolins@une.edu

## Slide 2
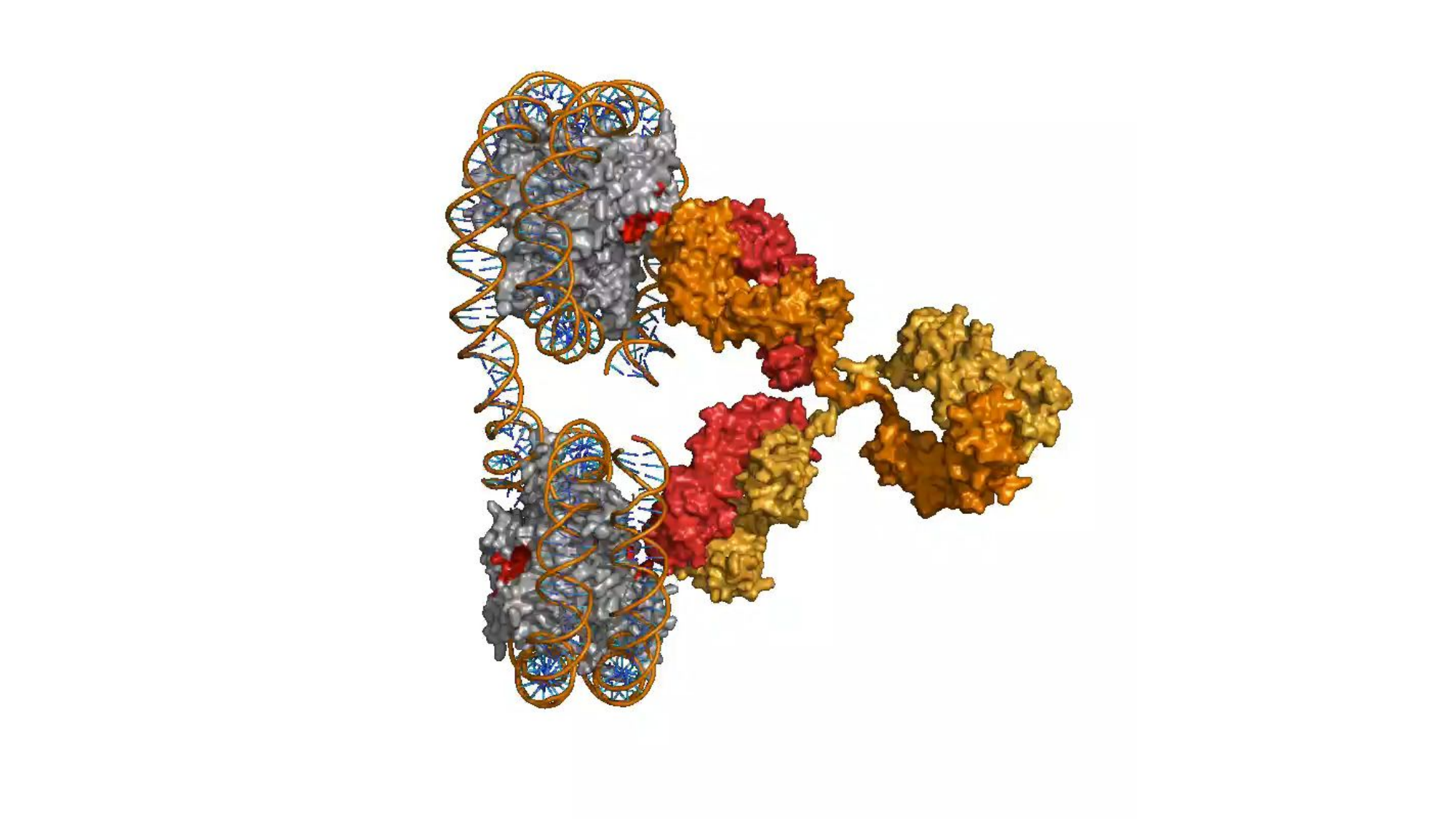

Supplement: Possible binding of Antibody PL2-6 to a Dinucleosome [file rsob180058supp1.pptx]
